# Spontaneous Multiple Knotting of a Feeding Tube Urinary Catheter in an Infant with Crouzon Syndrome: A Case Report

**DOI:** 10.3390/reports9020165

**Published:** 2026-05-22

**Authors:** Konstantinos Gkialas, Anna Papakonstantinou, Dimitrios Deligiannis, Aris Kaltsas, Panagiotis Mitsos

**Affiliations:** 1 Department of Urology, General Children’s Hospital “Agia Sofia”, Thivon 1 & Papadiamantopoulou, 11527 Athens, Greece; 2 Third Department of Urology, Attikon University Hospital, School of Medicine, National and Kapodistrian University of Athens, 12462 Athens, Greece

**Keywords:** Crouzon syndrome, urinary catheter, feeding tube, catheter knotting, infant, cystotomy

## Abstract

**Background and clinical significance**: Catheter knotting is a rare but potentially serious complication of urethral catheterization in neonates and infants, particularly when feeding tubes are used due to small urethral caliber. **Case Presentation**: We report the case of a 6-month-old male infant with Crouzon syndrome who underwent cranioplasty. Intraoperatively, a 6 Fr feeding tube was inserted for bladder drainage. On postoperative day 6, resistance was encountered during catheter removal. Radiography revealed a double knot in the distal urethra and a single knot in the proximal urethra. The catheter was successfully removed surgically via cystotomy, and the infant recovered uneventfully with normal voiding function. **Conclusions**: This case demonstrates the exceptional occurrence of simultaneous double and single knots in a urinary catheter. Awareness of this rare complication, careful control of insertion length, and prompt intervention upon resistance are essential to prevent urethral trauma and ensure patient safety.

## 1. Introduction and Clinical Significance

In neonates and young infants, catheterization presents unique technical challenges owing to the small caliber of the urethra, limited bladder capacity, and the frequent need to substitute standard Foley catheters with alternative devices such as feeding tubes. Although the procedure is generally considered safe, rare but potentially serious complications have been described in the pediatric population. Among these complications, spontaneous knotting of a urinary catheter is particularly uncommon but clinically significant. The reported incidence is extremely low, estimated at approximately 0.2 per 100,000 catheterizations in children [1,2,3]. The phenomenon occurs predominantly in neonates and infants and is most often associated with the use of long, highly flexible catheters inserted excessively into the bladder. The most widely accepted mechanism involves overinsertion of a flexible catheter into the bladder, allowing it to coil and form loops; the catheter tip may subsequently pass through a loop, resulting in knot formation. The formation of a knot may result in resistance during catheter removal and can lead to urethral trauma, mucosal injury, bleeding, infection, or, in severe cases, the need for surgical intervention [4]. Identified risk factors include excessive insertion length, use of small-caliber and highly flexible catheters (such as feeding tubes), and inadequate fixation. Prevention strategies focus on limiting insertion depth to the minimum required for urine drainage, using appropriately sized catheters, and securing the catheter to avoid migration. The majority of cases reported in the literature describe the formation of a single intravesical knot. The occurrence of multiple knots in a single catheter is exceptionally rare, with only isolated reports documenting double knot formation [5]. The simultaneous presence of both a double knot and a single knot within the urinary tract is exceedingly uncommon and represents a unique and challenging clinical scenario. Management depends on knot complexity and ranges from gentle traction and minimally invasive techniques (e.g., guidewire-assisted removal or fluoroscopic manipulation) to open surgical intervention when conservative approaches fail.

Crouzon syndrome is a rare autosomal dominant craniosynostosis disorder caused by mutations in the FGFR2 gene, characterized by premature fusion of cranial sutures and distinctive craniofacial abnormalities [6,7]. The estimated incidence of Crouzon syndrome ranges from 1 in 25,000 to 1 in 60,000 live births. Although not directly associated with urological abnormalities, affected infants frequently undergo complex surgical procedures and require intensive care, during which urinary catheterization is commonly performed.

Diagnosis is established through a combination of clinical assessment, radiologic imaging demonstrating premature suture fusion, and confirmatory molecular genetic testing [8]. Management requires a multidisciplinary approach involving craniofacial and neurosurgical teams, as well as specialists in ophthalmology, otolaryngology, orthodontics, and medical genetics [8]. Surgical treatment is typically staged and aims to relieve intracranial hypertension, preserve visual function, improve airway patency, and correct craniofacial deformities [9]. Interventions may include early cranial vault remodeling followed by midface advancement during later childhood or adolescence [7]. With early diagnosis, structured follow-up, and timely surgical intervention, long-term functional and aesthetic outcomes are generally favorable, although many patients require multiple reconstructive procedures throughout growth and development [8].

In this report, we present a rare and unusual case of a 6-month-old male infant with Crouzon syndrome who developed spontaneous formation of both a double knot and a single knot in a feeding-tube-type urinary catheter following craniofacial reconstructive surgery.

## 2. Case Presentation

A 6-month-old male infant with a known diagnosis of Crouzon syndrome was admitted to the Pediatric Neurosurgical Department for elective craniofacial reconstructive surgery. The infant underwent cranioplasty under general anesthesia. Standard anesthetic monitoring was applied, and the procedure proceeded without intraoperative complications. For perioperative urine output monitoring and bladder decompression, a soft, flexible 6 Fr feeding tube was used as a urinary catheter, as the small urethral caliber in infants often precludes the use of standard balloon (Foley) catheters. The catheter was inserted transurethrally without resistance, urine output was promptly observed, and correct placement was presumed. The estimated length of the catheter advanced into the bladder was approximately 25 cm, exceeding the recommended insertion depth for infants and potentially contributing to intravesical coiling and knot formation. The catheter was secured in place and left indwelling at the conclusion of the procedure.

Following surgery, the infant was transferred to the Neonatal Intensive Care Unit (NICU) for close postoperative monitoring and supportive care. The immediate postoperative period was stable, with no complications. The urinary catheter remained in place for continuous bladder drainage during the NICU stay. During the postoperative period, intermittent urine leakage around the catheter was observed, indicating suboptimal drainage. Despite this, urine output was maintained, and there was no evidence of urinary retention or bladder distension. The patient remained hemodynamically stable, with no clinical or laboratory signs of renal impairment. Due to the catheter dysfunction, removal and replacement were planned.

On postoperative day 6, removal of the urinary catheter was attempted. Gentle traction was applied, but resistance was encountered, and the catheter could not be withdrawn. Multiple careful attempts were made to reposition and manipulate the catheter, but removal remained unsuccessful. Importantly, these attempts did not result in urethral bleeding, hematuria, or signs of acute distress. Given the unexpected resistance and concern for a mechanical complication, further attempts at removal were abandoned.

**Figure 1:**
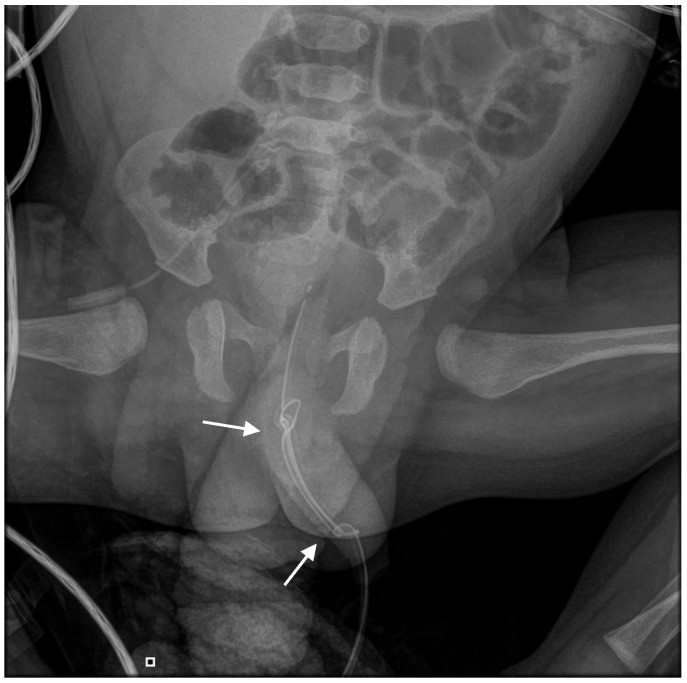
X-ray of the lower abdomen of the patient, showing the double knot located at the distal urethra and the single knot located at the proximal urethra (near the bladder neck) of the urinary catheter (the arrows are pointing at the knots).

An abdominal radiograph was obtained to evaluate the position and configuration of the catheter. Imaging revealed an abnormal appearance of the catheter with evidence of knot formation. Specifically, the radiograph demonstrated a double knot located distally within the urethra and a separate single knot situated more proximally, near the bladder neck (Figure 1).

**Figure 2:**
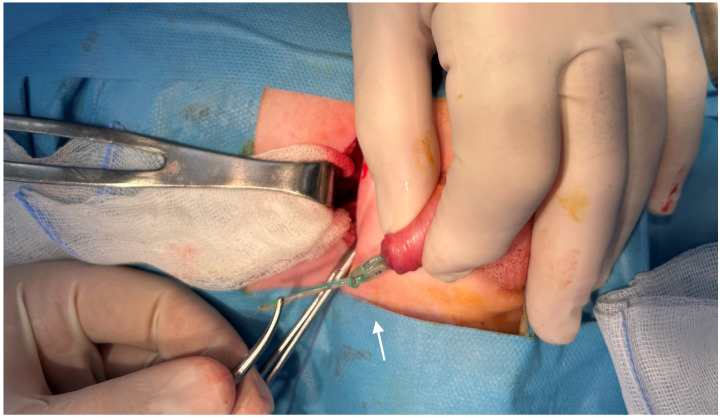
The distal part of the urinary catheter, featuring the double knot (arrow).

At this point, conservative removal was deemed unsafe due to the risk of urethral trauma or mucosal laceration. The patient underwent surgical management under general anesthesia. A Pfannenstiel incision was performed to access the bladder extraperitoneally. A small cystotomy was carefully created, allowing direct visualization of the intravesical portion of the catheter. The proximal single knot was identified at the level of the bladder neck and was gently mobilized using mosquito forceps. Attention was then directed to the distal portion of the catheter (Figure 2).

**Figure 3:**
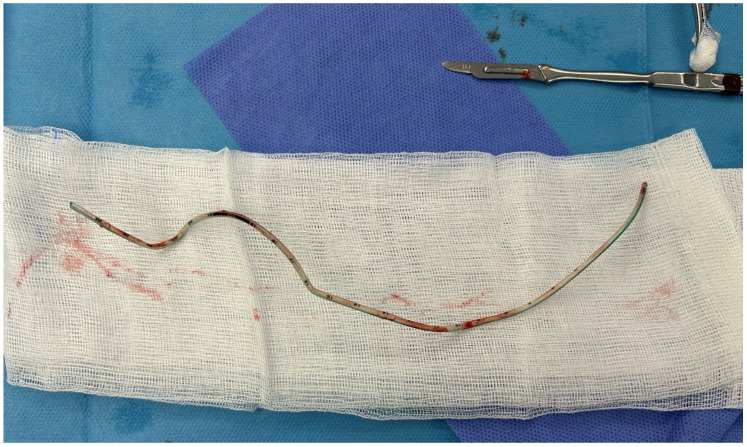
The feeding tube urethral catheter following removal after surgical division.

The double knot was sequentially loosened and untied, beginning distally and progressing proximally, to facilitate safe extraction. Once the knots were successfully undone, the catheter was removed through the cystotomy without resistance (Figure 3).

The bladder was subsequently repaired in a standard layered fashion. An attempt to place a transurethral urinary catheter postoperatively was unsuccessful due to urethral edema. Consequently, a suprapubic catheter was placed to ensure adequate urinary drainage, along with a Penrose drain for postoperative monitoring.

Postoperatively, the infant recovered uneventfully in NICU, with stable vital signs and adequate urine output. The suprapubic catheter was removed ten days after surgery without difficulty. Following catheter removal, the infant demonstrated normal spontaneous voiding with no evidence of hematuria, urinary retention, or infection. The Penrose drain was removed on postoperative day eleven after minimal output was observed. At 6-month follow-up, the infant is clinically well, with normal spontaneous voiding, a good urinary stream, and a normal renal and bladder ultrasound, with no evidence of urethral stricture or other urological complications.

## 3. Discussion

The use of urinary catheters is one of the most frequently performed invasive procedures in pediatric medical and surgical care. Urethral catheterization is generally considered safe and is commonly carried out in infants and children. As the urethra of neonates and small infants is too narrow for the smallest available Foley catheter (6 Fr), feeding tubes are often used as practical alternatives for bladder drainage. These tubes are readily available, cost-effective, and sufficiently rigid for use in small-caliber urethras [10]. However, their increased flexibility and length may predispose to rare complications, including catheter knotting, particularly in neonates and infants with small bladder volumes [11,12].

Although spontaneous catheter knotting is rare, cases have been reported in the literature since the first description in 1976 [13], including early case series describing up to five patients and more recent reports [5,14]. Catheter knots have been estimated to occur in 0.2 per 100,000 catheterizations in children [12,15]. Knotting of urinary catheters has been observed more frequently in males than in females, and more commonly in neonates and children than in adults [16]. The most likely mechanism involves insertion of an excessively long segment of the feeding tube into the bladder. The redundant length allows the catheter to coil upon itself as it contacts the bladder wall, permitting the distal tip to pass through an open loop and form a knot [17].

In the present case, the estimated insertion length (~25 cm) far exceeded the recommended depth for infants (~6 cm), resulting in substantial intravesical redundancy. This excessive length likely facilitated repeated coiling of the catheter within the bladder, allowing the tip to pass through multiple loops and form complex knot configurations. The high flexibility and smooth surface of the feeding tube may have further promoted loop formation and tightening, contributing to the simultaneous presence of both a double knot and a separate single knot.

Spontaneous knotting of urinary catheters is an uncommon but clinically significant complication in pediatric practice, occurring predominantly in neonates and young infants whose anatomical characteristics predispose them to mechanical adverse events [16]. The limited bladder capacity, narrow urethral lumen, and increased tissue fragility in this age group create conditions under which even minor technical deviations may result in disproportionate consequences. The present case is, to our knowledge, the first documented manifestation of the concurrent formation of a double knot and a single knot in a feeding-tube-type urinary catheter in a 6-month-old infant with Crouzon syndrome, adding to the scarce body of literature on complex catheter knotting in children.

Carlson and Mowery tabulated the appropriate catheter size and depth of insertion according to age and sex in pediatric patients, providing practical guidance aimed at minimizing catheter-related complications [18]. Specifically, they recommend the use of 5–8 Fr catheters with an insertion depth of approximately 6 cm in infant boys [18,19]. Adherence to these recommendations is especially important in neonates and young infants, as excessive catheter insertion length may increase the risk of intravesical looping and knot formation. Prevention of catheter knotting is closely related to proper insertion technique. The catheter should be advanced only until urine flow is obtained, followed by minimal additional advancement to ensure adequate positioning. In neonates and infants, recommended insertion depths are typically limited to approximately 4–6 cm, and exceeding this range may significantly increase the risk of intravesical coiling and subsequent knot formation [20].

As a result, feeding tubes continue to be commonly used as alternatives for urinary bladder drainage, especially in neonatal and pediatric intensive care units [19]. While feeding tubes are readily accessible and easy to insert, their increased flexibility, smooth surface, and excessive length relative to bladder capacity significantly increase the risk of intravesical coiling and knotting. Advancing excessive catheter length significantly increases the risk of loop formation and knotting. To prevent knot formation, it is recommended that only a minimal length of tubing be advanced into the bladder—just sufficient to ensure adequate drainage—and that the catheter be secured in place with adhesive tape [18,19]. In most reported cases, knotted catheters can be removed using non-operative techniques, including gentle traction, guidewire assistance, or fluoroscopic manipulation [11]. However, in complex or refractory cases—particularly when multiple knots are present or resistance is significant—open surgical intervention may be necessary to safely remove the catheter and prevent urethral or bladder injury [11].

Vigilance during all phases of catheter management is essential. Healthcare professionals involved in catheter insertion, maintenance, and removal should remain vigilant, particularly when resistance is encountered during catheter removal, as early recognition is critical to prevent urethral trauma. Resistance should be regarded as a warning sign of potential mechanical entanglement [16]. Further traction should be avoided, as forceful extraction may result in urethral mucosal injury, bleeding, false passage formation, or long-term complications such as urethral stricture. Early recognition and prompt diagnostic evaluation, most commonly with plain radiographic imaging, are critical steps in confirming the diagnosis and guiding safe management [21].

Preventive strategies include selecting appropriately sized catheters and limiting insertion length according to the child’s age and sex. The use of feeding tubes for urinary catheterization should be avoided whenever possible, as their excessive flexibility and length substantially increase the risk of looping and knot formation [22]. Balloon-tipped urethral catheters are preferable when available, as they reduce the risk of knotting [21]. In cases where knotting occurs and conservative removal fails, prompt urological evaluation and timely surgical intervention are essential to ensure patient safety.

## 4. Conclusions

Catheter knotting is a rare complication of urethral catheterization. Although multiple cases of urethral catheter knotting have been reported in the literature, the simultaneous presence of multiple knots (double and single knot) on a urethral catheter in an infant appears to be exceptionally rare. With proper training and adherence to recommended insertion techniques, the likelihood of this rare but hazardous complication can be minimized. Ultimately, this case serves as a reminder that even routine procedures demand meticulous technique and sustained clinical awareness to ensure patient safety and optimize outcomes in vulnerable pediatric populations.

## Data Availability

The original data presented in this study are available on reasonable request from the corresponding author. The data are not publicly available due to privacy concerns.

## Abbreviations

The following abbreviations are used in this manuscript:

NICU	Neonatal Intensive Care Unit
FGFR2	Fibroblast Growth Factor Receptor 2
